# The Association between Breakfast Skipping and Body Weight, Nutrient Intake, and Metabolic Measures among Participants with Metabolic Syndrome

**DOI:** 10.3390/nu9040384

**Published:** 2017-04-14

**Authors:** Lijuan Zhang, Lorraine S. Cordeiro, Jinghua Liu, Yunsheng Ma

**Affiliations:** 1 Department of Cardiology, Key Laboratory of Arrhythmias, Ministry of Education, Shanghai East Hospital, Tongji University School of Medicine, Tongji University, Shanghai 200120, China; zhangxiaoyi@tongji.edu.cn; 2 Division of Preventive and Behavioral Medicine, Department of Medicine, University of Massachusetts Medical School, Worcester, MA 01655, USA; 3 Department of Nutrition, School of Public Health, University of Massachusetts Amherst, Amherst, MA 01003, USA; lcordeiro@nutrition.umass.edu; 4 Massachusetts Supranational TB Reference Laboratory, Commonwealth Medicine, University of Massachusetts Medical School, Worcester, MA 01605, USA; jinghua.liu@umassmed.edu

**Keywords:** breakfast skipping, weight loss, nutrient intake, metabolic syndrome

## Abstract

The effect of skipping breakfast on health, especially in adults, remains a controversial topic. A secondary data analysis was conducted to examine associations between breakfast eating patterns and weight loss, nutrient intake, and metabolic parameters among participants with metabolic syndrome (MetS) (*n* = 240). Three randomly selected 24-h dietary recalls were collected from each participant at baseline and at the one-year visit. Skipped breakfast was seen in 32.9% at baseline and in 17.4% at the one-year visit, respectively. At baseline, after adjustment for demographics and physical activity, participants who ate breakfast had a higher thiamin, niacin, and folate intake than did breakfast skippers (*p* < 0.05); other selected parameters including body weight, dietary quality scores, nutrient intake, and metabolic parameters showed no significant differences between the two groups (*p* ≥ 0.05). From baseline to one year, after adjustment for covariates, mean fat intake increased by 2.7% (95% confidence intervals (CI): −1.0, 6.5%) of total energy in breakfast skippers in comparison to the 1.2% decrease observed in breakfast eaters (95% CI: −3.4, 1.1%) (*p* = 0.02). Mean changes in other selected parameters showed no significant differences between breakfast skippers and eaters (*p* > 0.05). This study did not support the hypothesis that skipping breakfast has impact on body weight, nutrient intakes, and selected metabolic measures in participants with MetS.

## 1. Introduction

Breakfast skipping continues to be a controversial public health issue. Current evidence does not support a clear effect of regularly consuming or skipping breakfast on body mass/composition, nutrient intake, or metabolic parameters. Some studies suggest that breakfast is the most important meal of the day, with consumption associated with a higher calcium and fiber intake and, consequently, a lower body mass index [1]. A series of studies have reported that breakfast skipping is associated with obesity [2], hypertension [3] cardiometabolic disease [4], lower dietary quality scores [5], insulin insensitivity, and diabetes mellitus [6] and mortality [7]. In addition, studies examining vitamin and mineral intake (i.e., fiber, B_6_ and B_12_, niacin, riboflavin, thiamin, vitamins A and C, calcium, iron, magnesium, phosphorus, potassium, and zinc) related to breakfast skipping showed that eating a nutrient-dense breakfast had more favorable effects on weight loss and nutrient intake than breakfast skipping [8,9,10]. However, several previous studies did not find any association between breakfast eating patterns and weight loss [11,12,13,14,15,16]; some studies even indicated that skipping breakfast led to weight loss [17,18,19]. A four-year cohort study based on Japanese insurance statistics indicated that self-reported breakfast skippers had a lower incidence of all diseases, including metabolic diseases, as compared to breakfast eaters [20]. Mattson and colleagues (2003) showed that the effect of breakfast skipping was similar to intermittent fasting resulting in reduction of caloric intake, which would have metabolic benefits [21].

In 2011, a comprehensive review by the US Department of Agriculture (USDA) indicated an increased risk of overweight and obesity in children and adolescents who skipped breakfast, but in adults, the findings were inconsistent [22]. The conclusion of this review was supported by a systematic review inclusive of 153 articles published from 2008 to 2010. The review also noted the limitation that most studies reporting increased risk of overweight and obesity in children and adolescents who skipped breakfast did not control for some potential confounders such as dietary quality and physical activity [23]. Previous studies concluded that skipping breakfast may not be enough to alter weight and most health outcomes in adults [11,24]. Betts et al. (2014) found that health indexes were unaffected by breakfast skipping in lean adults [25]. Geliebter et al. (2014) found that skipping breakfast can lead to weight loss [17], possibly due to a reduction in daily energy intake in some adults [26]. A cumulative meta-analysis performed by Brown et al. (2013) indicated that the observational studies on the effect of breakfast skipping on obesity has gratuitously established the association, but not the causal relationship [27].

Using a secondary data analysis from a one-year dietary interventional trial, our current study investigated the association between breakfast skipping and body weight, nutrient intake, and metabolic parameters in participants with metabolic syndrome (MetS).

## 2. Methods

### 2.1. Study Design and Sample

The dietary intervention trial was conducted from June 2009 to January 2014 (ClinicalTrial.gov: NCT00911885). Full details of the study methodology have been described elsewhere [28]. Briefly, individuals were recruited in 10 waves between June 2009 and January 2012 from Worcester, Massachusetts, United States. Inclusion criteria were age between 21–70 years, a body mass index (BMI) of 30–40 kg/m^2^, nonsmokers, and a diagnosis of MetS [29]. Exclusion criteria were clinically diagnosed diabetes or fasting blood sugar levels ≥126 mg/dL, an acute coronary event within the last six months, pregnant or lactating, polycystic ovary syndrome, intention to move out of the area within the one-year study period, current participation in a weight loss program, an eating disorder, bariatric surgery, elevated depression or suicidal ideation, or subjects following a low-carbohydrate, high fat dietary regimen.

A total of 240 eligible participants with MetS were enrolled into the study and randomized to one of two conditions: (1) the American Heart Association (AHA) diet (*n* = 119) or, (2) a high fiber diet (*n* = 121). Each participant attended 2 individual sessions and 12 group sessions during the 1-year counselling period for his/her assigned diet. However, participants had not been given any intervention for breakfast eating pattern during the period of the trial. Six participants did not report their breakfast eating pattern at baseline. At the one-year visit, a total of 178 participants completed breakfast status reports and were included in the data analysis. The study protocol was approved by the Institutional Review Board (IRB) at the University of Massachusetts Medical School.

### 2.2. Assessment of Dietary Intake

Three 24 h dietary recalls on randomly selected days, including two weekdays and one weekend day, were used to evaluate dietary intake at each visit (within a three week window) [30]. Dietary recalls were conducted over the telephone by a registered dietitian not associated with intervention, and participants were provided with two-dimensional food portion models before the call. Twenty four–hour physical activity recalls were administered after the diet recalls. Dietary intake data were analyzed using the Nutritional Data System for Research (NDS-R, 2010–2012, Minneapolis, MN, USA) [31]. Dietary quality was measured using the Alternative Healthy Eating Index (AHEI), an instrument designed to evaluate nine criteria of a healthy cardiovascular diet [32]. All individual component scores were summed for a total AHEI score ranging from 2.5 (worst) to 87.5 (best). Calculation of AHEI scores followed the same methodology detailed in our previous publication [33].

### 2.3. Breakfast Assessment and Definitions

Although there are 11 definitions of eating breakfast [34], few differences were found between self-reported eating breakfast and other definitions [35]. In this study, breakfast consumption was self-reported and included consumption of any food/beverage (excluding water) at the first eating episode no later than 10.00 a.m. Three unannounced 24 h dietary recalls were used to create the eating pattern variables of interest. Participants were asked to report in detail all foods and beverages consumed in the 24 h preceding the dietary interview. Detailed time, meal location, and parallel activities were also recorded for each eating occasion. According to their three 24-h dietary recalls, subjects who reported eating breakfast at least once were considered as breakfast eaters and those who did not consume any breakfast on the three dietary recall days were categorized as breakfast skippers. At baseline, 32.6% of subjects reported they ate breakfast every day, 13.8% ate breakfast on two of the recall days, 20.7% ate breakfast on one of the recall days, and 32.9% ate nothing for breakfast every day.

### 2.4. Other Variables of Interest

Height was measured at baseline only, and weight was measured at each time point using a balance scale (Detecto 339 Model scale; Webb City, MO, USA). Participants wore light clothes and removed shoes for height and weight measurements. BMI (kg/m^2^) was calculated using the value of weight in kilogram divided by height in meter squared. Waist circumference was measured twice at the narrowest part of the torso (i.e., a site between the lower rib and crest of the hipbone). All anthropometric measurements were performed by trained researchers according to standardized techniques. Demographic data were collected using a self-administered questionnaire at baseline. Blood pressure was measured using a sphygmomanometer with the subject in a sitting position. Hypertension was defined as a systolic blood pressure ≥140 mm Hg and/or a diastolic blood pressure ≥90 mm Hg [36]. Laboratory data consisted of total cholesterol (TC), high density lipoprotein cholesterol (HDL-C), low density lipoprotein cholesterol (LDL-C), triglyceride, hemoglobin A1c (HbA1c), fasting glucose level (FG), and fasting plasma insulin. Glucose and insulin were measured using fasting blood samples [37]. Homeostasis Model Assessment of Insulin Resistance (HOMA-IR) score was calculated as, HOMA-IR = (glucose) × (insulin)/405 (glucose in mg/dL, Insulin in mU/L). 

### 2.5. Statistical Analyses

A secondary data analysis was conducted to examine the association between breakfast skipping and body weight, nutrient intake and selected metabolic parameters. Since there was not a statistically significant difference between AHA diet and high-fiber diet conditions in weight loss (mean group difference, 0.4 kg (95% CI, −0.7 to 1.5 kg) [33]), the two groups were combined to increase statistical power to examine the effect of breakfast skipping on body weight and other parameters. Since there were only 36 subjects who changed their breakfast eating patterns over the one year period (eight participants changed from breakfast eaters to skippers and 28 participants changed from breakfast skippers to eaters), there was not a sufficient sample size for comparative data analysis. As such, they were removed from further prospective analysis.

All statistical analyses were performed by using SAS 9.4 (SAS Institute, Cary, NC, USA). Values were presented as means (95% confidence interval (CI)) for continuous variables and *n* (%) for categorical variables. Comparisons between groups for baseline characteristics were conducted by using Chi-square tests or fisher’s exact tests. For data that were right-skewed including FG, insulin, HOMA-IR, TC, HDL-C, LDL-C, and triglyceride, log-transformation was used. Analyses for added sugar, protein, fat, fiber, and carbohydrate (% of energy) were performed after adjusting for total energy intake. General linear regression model analysis was used to examine the associations between breakfast skipping and body weight, nutrient intake, and metabolic parameters. Multivariable regression modeling was used to adjust for covariates, including sex, age, race, educational level, study condition and physical activity. Total energy intake was included in the models when assessing physical characteristics and metabolic parameters. *p*-value of 0.05 was considered statistically significant.

## 3. Results

### 3.1. Characteristics of Participants

About 32.9% of participants skipped breakfast at baseline, and 17.4% at the one-year visit. The majority of participants (88.4%) were white, 26.9% were male, the mean age was 52.2 years old, and the mean BMI for men and women was 34.8 and 35.1 kg/m^2^, respectively. 

Table 1 shows that between breakfast skippers and eaters, no meaningful difference was observed on study condition (AHA and high fiber), gender, age groups, educational level, BMI, race/ethnicity, household income, and current work status at baseline (*p* > 0.05). At baseline, mean duration of total physical activity was 235.1 min/week (95% CI: 208.5, 261.7 min/week) for breakfast skippers, much higher than that (200.4 min/week, 95% CI: 184.8, 216.0 min/week) for breakfast eaters (*p* = 0.02), but this difference was attenuated at the one year visit (*p* = 0.16).

### 3.2. Dietary Nutrient Intake and Metabolic Parameters

A detailed unadjusted and multivariate adjusted means of physical characteristics, dietary nutrient intake, and selected metabolic parameters at baseline in breakfast skippers and eaters are presented in Table 2. Total energy intake was 1757.3 kcal/day (95% CI: 1614.9, 1899.8 kcal/day) in breakfast skippers, much lower than the 1945.3 kcal/day (95% CI: 1845.6, 2045.1 kcal/day) in breakfast eaters (*p* = 0.03), which was attenuated after adjustment for age, gender, race, educational level, study condition and total physical activity (*p* = 0.05). The same phenomena were found in total cholesterol, sodium and potassium intake. Skipping breakfast was associated with a significantly lower thiamin, niacin and folate intake even in an adjusted multivariate model at baseline. In an unadjusted model, daily folate intake was 371.7 µg (95% CI: 337.2, 406.1 µg) in breakfast skippers, lower than 417.0 µg (95% CI: 392.9, 441.2 µg) in breakfast eaters (*p* = 0.03). The corresponding values were 358.3 µg (95% CI: 312.6, 403.8 µg) versus 406.4 µg (95% CI: 371.8, 441.0 µg) in an adjusted multivariate model (*p* = 0.02). Daily niacin intake was 21.2 mg (95% CI: 19.4, 23.0 mg) in breakfast skippers, lower than the 23.9 mg (95% CI: 22.7, 25.1 mg) in breakfast eaters (*p* = 0.01) in an unadjusted model. The corresponding values were 21.4 mg (95% CI: 19.2, 23.7 mg) versus 23.7 mg (95% CI: 22.0, 25.4 mg) in an adjusted multivariate model (*p* = 0.02). The lower daily thiamin intake was also detected in breakfast skippers in both unadjusted and adjusted multivariate models (*p* = 0.01). No statistically significant differences in physical characteristics, dietary quality, and selected metabolic parameters were found between breakfast skippers and eaters (*p* ≥ 0.05).

### 3.3. Changes of Dietary Nutrient Intake and Metabolic Parameters from Baseline to One Year

Table 3 indicated that mean change in total fat intake as a percentage of total energy in breakfast skippers was 2.7% (95% CI: −1.0, 6.5%), much higher than the −1.2% (95% CI: −3.4, 1.1%) in breakfast eaters in an adjusted multivariate model (*p* = 0.02). Mean changes in other selected parameters showed no significant differences between breakfast skippers and eaters in both unadjusted and adjusted models (*p* ≥ 0.05).

## 4. Discussion

It is widely believed that breakfast skipping leads to weight gain, yet there is limited consensus as to the association [27]. One limitation is the lack of a standard breakfast definition, which might account for conflicting research results [38]; another limitation is the uncertain hypothesis that skipping breakfast leads to lower satiety than if breakfast had been consumed, thus overeating will ensue later in the day, which over time would result in weight gain [27]; additionally, lifestyle characteristics that affect weight maintenance such as physical activity and nutrient intake had not been considered in most studies [23]. In this study, we used self-reported data to define breakfast skipping and breakfast consumption, which was in close agreement with a well-defined definition (Skinner’s definition [39]). Furthermore, self-reports appeared to be the most convenient method to process eating pattern data in epidemiological studies [35]. For understanding whether breakfast skippers consumed more calories later in the day, we compared the daily energy intake between breakfast skippers and eaters. Furthermore, numerous potential confounders including sociodemographic variables, daily energy intake and physical activity were adjusted in our multivariable regression models. In contrast to previous studies reporting that breakfast skipping was associated with lower levels of physical activity [40,41], these results showed that breakfast skippers reported more exercise at baseline. Skipping breakfast may reflect dieting patterns in overweight persons who are trying to control their weight [23].

### 4.1. Breakfast Consumption and Weight Loss

This study is among the first to systematically examine the associations between breakfast skipping and body weight, dietary intake, and metabolic parameters among participants with MetS aged 21–70 years using a cross-sectional as well as a one-year prospective longitudinal analysis. Based on self-reports, 32.9% of subjects reported skipping their breakfast at baseline, which was much higher than a 13.3% prevalence of breakfast skippers reported among American adults [42]. The difference in results could be explained by the special subjects among whom the studies were conducted. Previous studies have shown that skipping breakfast may increase the risk of metabolic syndrome [10,43], therefore, high percentages of breakfast skippers could be detected in subjects with MetS. The percentage of breakfast skippers decreased to 17.4% at the one-year follow-up visit, suggesting that participants had the intention to change their eating patterns when they participated in a dietary interventional trial.

Inconsistent with popular beliefs, these results did not show that breakfast skipping was consistently associated with weight gain, which was in agreement with the results of a randomized controlled trial performed with overweight and obese adults aged 20–65 years [11]. In order to understand the possible underlying reasons, this study further investigated daily energy and nutrient intake among these subjects.

### 4.2. Energy and Nutrient Intakes

Contrary to the hypothesis described above, daily total energy intake did not increase following the behavior of skipping breakfast, suggesting that overeating did not occur later in the breakfast-skipping day. This might explain, in part, why skipping breakfast had no significant effect on weight gain. Furthermore, Levitsky and Pacanowski (2013) suggest that skipping breakfast may be an effective means to reduce daily energy intake in some adults [26].

The results regarding the quality of diet disagree with the findings of several studies [5,44,45]. Previous studies have reported that breakfast skippers are more likely to have unhealthy dietary habits. Breakfast eaters usually consume larger amounts of fiber and lower of total fat and sugar [46]. No significant differences were detected among these parameters including dietary quality between breakfast eaters and skippers at both baseline and the one-year visit in our study, which indicated that diet quality may be affected by breakfast type [47]. In our study, in good agreement with the previous cross-sectional studies, breakfast skippers had a lower daily thiamin, niacin, and folate intake [26,48]. However, the average intakes of thiamin and niacin among these subjects in this study were still a little higher than the recommended intakes by the USDA [49], indicating that these subjects could consume enough of these two nutrients from other meal types to supply their daily requirements. For average daily folate intake, breakfast skippers consumed less than the 400 µg recommended by USDA [49] at baseline and the one-year visit, which implied that breakfast might be the main food sources of folate [50]. Folate deficiency is associated with elevated plasma levels of homocysteine, a risk factor for occlusive cardiovascular disease [51], so consumption of folate containing foods is an important dietary component for breakfast skippers. Considering the limitation of the cross-sectional design, this study further evaluated the daily nutrients intake using a one-year prospective analysis and the results showed no significant differences in changes in nutrient intakes between breakfast skippers and breakfast eaters.

### 4.3. Metabolic Parameters

Moderate evidence supports a positive association between the behavior of breakfast consumption and cardiometabolic risk factors [4] and diabetes [52] in children and adolescents. A large prospective study with Australians aged 26–36 years found that breakfast skipping during childhood and teenage years led to adverse changes in waist circumference, fasting insulin levels, total and LDL cholesterol levels [53]. In contrast, research on Korean adults, aged 30–50 years, found that breakfast skipping was associated with a reduced risk of elevated serum triglyceride levels [10]. In this study, no statistically significant differences in blood pressure and metabolic parameters were found between breakfast skippers and eaters among participants with MetS. In contrast to this study’s findings, Chung and colleagues (2015) reported the odds ratios for the risk of obesity and metabolic syndrome increased in breakfast skipping participants aged 20–64 years due to poor daily nutrient intake with a high percentage of energy from fat [48]. The difference between this study’s results and those of Chung et al. might be that they compared the breakfast skipping group across various specific breakfast types such as consumption of rice, noodles, or cereal groups [48]. This study’s findings regarding cardiometabolic risk factors are further supported by the results of a randomized controlled trial [54]. In that study, advising a prudent breakfast for three months did not influence blood lipids, body weight, or glucose metabolism among participants aged 25–67 years [54]. Similarly, no differences in cardiometabolic measurements were found across breakfast consumption patterns in 51–70 year old adults by McGill and colleagues (2013) [55]. Cardiometabolic measures were consistently higher in older compared to younger adults, particularly among those with MetS [56]; this could prevent modest effects of diet from being observed. Additionally, participants with MetS may be more likely to use anti-hypertensive or lipid-lowering medications, so diet may have less of an impact [57].

The current investigation had several strengths. It was the first study to examine associations between breakfast skipping and weight loss in adult participants with MetS using both cross-sectional and longitudinal analyses. This study measured a wide range of dietary and metabolic parameters in order to assess healthy effects related to breakfast eating patterns.

This study had several limitations. First, it was a secondary data analysis based on a clinical trial, and the sample size was not truly representative of the general MetS population. Second, although it provided both descriptive and analytic studies, limitations of observational data such as self-reporting bias, potential confounders and interpretations about the results might also exist. Third, some individuals in this investigation did not report consistent food records; therefore, interpretations about dieting practices, such as breakfast skipping and micronutrient intake, may have inaccuracies. Last, although participants were not given any breakfast eating intervention during the period of trial, psychology-dependent changes due to joining a nutrition intervention trial might influence eating patterns.

## 5. Conclusions

In summary, the present study indicates that skipping breakfast alone may not be sufficient to influence body weight, dietary nutrient intake, and selected metabolic parameters in participants with MetS. Further research should be conducted to assess whether more specific recommendations with regard to the types of meals or meal quantity might improve weight-loss outcomes.

## Figures and Tables

**Table 1:** Participants’ characteristics by breakfast eating behavior at baseline and one year.

Group	Breakfast Eating Pattern at Baseline		*p*	Breakfast Eating Pattern at One Year		*p*
	Skipping	Eating		Skipping	Eating	
Interventional groups, *n* (%)						
AHA group	44 (37.6)	73 (62.4)	0.13	17 (18.9)	73 (81.1)	0.60
High fiber group	33(28.2)	84 (71.8)		14 (15.9)	74 (84.1)	
BMI, *n* (%)						
30–34.9	35 (31.0)	78 (69.0)	0.54	19 (17.1)	92 (82.9)	0.71
35–40	42 (34.7)	79 (65.3)		12 (19.3)	50 (80.7)	
Gender, *n* (%)						
Male	16 (25.4)	47 (74.6)	0.14	7 (14.9)	40 (85.1)	0.60
Female	61 (35.7)	110 (64.3)		24 (18.3)	107 (81.7)	
Age, *n* (%)						
20–50 years	26 (28.3)	66 (71.7)	0.22	11 (19.0)	47 (81.0)	0.70
51–70 years	51 (35.9)	91 (64.1)		20 (16.7)	100 (83.3)	
Education, *n* (%)						
Bachelor’s degree or less	57 (32.8)	117 (67.2)	1.00	21 (16.2)	109 (83.8)	0.39
Graduate/professional	19 (32.8)	39 (67.2)		10 (21.7)	36 (78.3)	
Race, *n* (%)						
White	72 (34.8)	135 (65.2)	0.09	28 (17.6)	131 (82.4)	1.00 ^†^
Others	5 (18.5)	22 (81.5)		3(15.8)	16 (84.2)	
Household income, *n* (%)						
$0–$50,000	27 (42.9)	36 (57.1)	0.13	9 (18.3)	39 (81.2)	0.95
More than $50,001	33 (28.0)	85 (72.0)		15 (16.7)	75 (83.3)	
Unclear	17 (32.1)	36 (67.9)		7 (17.5)	33 (82.5)	
Currently working, *n* (%)						
Employed full-time or part-time	59 (32.2)	124 (67.8)	0.74	24 (17.4)	114 (82.6)	0.88
Others	17 (34.7)	32 (65.3)		7 (18.4)	31 (81.6)	
Mean duration of total physical activity, min/week	235.1 (208.5, 261.7)	200.4 (184.8, 216.0)	0.02	162.6 (119.9, 205.2)	197.8 (177.9, 217.7)	0.16

AHA: American Heart Association; BMI: body mass index. ^†^: Fisher’s Exact Test. Mean duration of total physical activity values are presented as mean 95% Confidence intervals (95% CI). Because of missing values, the total number of subjects differs.

**Table 2:** Relationship between breakfast eating pattern and physical characteristics and selected metabolic parameters at baseline.

	Breakfast Eating Pattern					
	Unadjusted			Multivariable Adjusted		
	Skipping	Eating	*p*	Skipping	Eating	*p*
Physical characteristics						
Body weight (lbs)	219.5 (212.8, 226.2)	217. 9 (213.2, 222.6)	0.70	231.9 (224.4, 239.3)	225.1 (219.4, 230.8)	0.05
Waist circumference (in)	40.2 (39.5, 41.0)	40.0 (39.5, 40.5)	0.64	41.2 (40.3, 42.1)	40.5 (39.9, 41.2)	0.09
BMI (kg/m^2^)	35.4 (34.7, 36.0)	34.8 (34.3, 35.3)	0.15	35.4 (34.4, 36.3)	34.7 (34.0, 35.4)	0.10
Blood pressure:						
Systolic (mmHg)	136.2 (134.0, 138.4)	135.5 (134.0, 137.1)	0.61	135.8 (132.8, 138.9)	134.9 (132.6, 137.2)	0.50
Diastolic (mmHg)	81.3 (79.4, 83.3)	79.9 (78.5, 81.3)	0.25	81.5 (78.8, 84.2)	79.5 (77.4, 81.6)	0.11
Daily dietary intake						
Total energy intake, kcal	1757.3 (1614.9, 1899.8)	1945.3 (1845.6, 2045,1)	0.03	1816.0 (1631.5, 2000.6)	1984.0 (1844.1, 2123.9)	0.05
Fat, %	32.5 (31.2, 33.8)	33.2 (32.3, 34.1)	0.38	31.8 (29.9, 33.6)	32.6 (31.2, 34.0)	0.30
Protein, %	17.6 (16.6, 18.5)	17.3 (16.7. 18.0)	0.71	17.4 (16.0, 18.8)	17.0 (16.0, 18.0)	0.52
Carbohydrate, %	47.5 (45.9, 49.2)	47.2 (46.1, 48.3)	0.74	49.0 (46.8, 51.2)	48.5 (46.9, 50.2)	0.65
Fiber, g	18.5 (17.0, 20.1)	19.4 (18.4, 20.5)	0.33	17.6 (15.6, 19.6)	18.8 (17.2, 20.3)	0.19
Cholesterol, mg	228.3 (199.5, 257.2)	277.6 (257.4, 297.8)	0.01	241.5 (206.5, 276.6)	269.5 (242.8, 296.2)	0.08
MUFA, %	11.5 (10.9, 12.0)	11.8 (11.4, 12.2)	0.33	11.5 (10.7, 12.3)	11.9 (11.3, 12.5)	0.30
PUFA, %	6.9 (6.4, 7.4)	7.2 (6.9, 7.6)	0.29	6.9 (6.2, 7.7)	7.3 (6.8, 7.9)	0.25
SFA, %	11.5 (10.9, 12.1)	11.4 (11.0, 11.8)	0.84	65.6 (53.0, 78.1)	70.9 (61.3, 80.4)	0.35
Added sugar, mg	54.3 (45.1, 63.4)	62.0 (55.6, 68.4)	0.17	65.6 (11.5, 15.2)	13.4 (13.4, 12.0)	1.00
Sodium, mg	2732.9 (2487.0, 2978.8)	3119.7 (2947.5, 3291.9)	0.01	2904.2 (2703.1, 3105.2)	3010.3 (2857.2, 3163.4)	0.25
Dietary quality scores	35.8 (33.4, 38.3)	37.6 (35.9, 39.3)	0.25	33.9 (30.5, 37.3)	36.3 (33.7, 38.9)	0.12
Daily intake of selected micronutrient						
Vitamin B_12_, µg	6.3 (4.7, 7.8)	4.8 (3.7, 5.9)	0.13	6.0 (3.8, 8.2)	4.5 (2.8, 6.2)	0.12
Vitamin B_6_, mg	1.7 (1.5, 1.8)	1.9 (1.8, 2.0)	0.04	1.7 (1.4, 1.9)	1.8 (1.7, 2.0)	0.05
Vitamin D, µg	4.1 (3.4, 4.9)	4.4 (3.9, 4.9)	0.60	4.0 (3.0, 5.0)	4.2 (3.4, 5.0)	0.62
Vitamin E, mg	12.0 (9.9, 14.1)	14.2 (12.7, 15.7)	0.10	11.5 (8.6, 14.5)	13.8 (11.6, 16.1)	0.08
Thiamin, mg	1.5 (1.4, 1.6)	1.7 (1.6, 1.8)	0.01	1.5 (1.3, 1.6)	1.7 (1.5, 1.8)	0.01
Calcium, mg	869.3 (776.7, 962.8)	927.2 (861.7, 992.7)	0.32	847.8 (720.9, 974.7)	901.9 (805.7, 998.1)	0.35
Magnesium, mg	276.9 (256.1, 297.8)	293.1 (278.5, 307.5)	0.21	265.0 (237.9, 292.1)	283.2 (262.6, 303.7)	0.14
Iron, mg	13.8 (12.5, 15.3)	15.2 (14.2, 16.1)	0.13	13.9 (12.0, 15.7)	15.0 (13.6, 16.4)	0.17
Zinc, mg	9.8 (8.9, 10.7)	11.1 (10.4, 11.7)	0.04	10.0 (8.7, 11.2)	11.0 (10.1, 12.0)	0.06
Niacin, mg	21.2 (19.4, 23.0)	23.9 (22.7, 25.1)	0.01	21.4 (19.2, 23.7)	23.7 (22.0, 25.4)	0.02
Folate, µg	371.7 (337.2, 406.1)	417.0 (392.9, 441.2)	0.03	358.2 (312.6, 403.8)	406.4 (371.8, 441.0)	0.02
Riboflavin, mg	2.0 (1.9, 2.2)	2.2 (2.1, 2.4)	0.10	2.0 (1.7, 2.2)	2.1 (2.0, 2.3)	0.11
Phosphorus mg	1153.7 (1061.1, 1246.3)	1250.6 (1185.7, 1315.4)	0.09	1126.7 (1006.6, 1246.7)	1221.8 (1130.7, 1312.8)	0.08
Potassium mg	2522.7 (2338.1, 2707.3)	2603.6 (2474.3, 2732.9)	0.48	2385.3 (2151.0, 2619.5)	2479.0 (2301.4, 2656.6)	0.38
Metabolic parameters						
HbA_1c_ level, %	5.7 (5.6, 5.8)	5.7 (5.6, 5.8)	0.98	5.7 (5.6, 5.9)	5.7 (5.6, 5.8)	0.91
Fasting glucose level, mg/dL ^†^	98.7 (96.0, 101.4)	98.4 (96.5, 100.3)	0.87	97.1 (93.5, 100.8)	97.4 (94.6, 100.2)	0.84
Fasting plasma insulin level, mU/L ^†^	10.0 (8.5, 11.8)	11.9 (10.5, 13.4)	0.10	11.5 (9.1, 14.5)	13.4 (11.2, 16.0)	0.16
HOMA-IR score ^†^	2.4 (2.1, 2.9)	2.9 (2.5, 3.3)	0.13	2.8 (2.2, 3.5)	3.2 (2.7, 3.9)	0.18
Blood lipid levels						
Total cholesterol level, mg/dL ^†^	192.7 (185.2, 200.4)	194.4 (189.1, 199.8)	0.72	183.1 (173.6, 193.1)	186.5 (179.1, 194.2)	0.45
HDL cholesterol level, mg/dL ^†^	47.5 (45.4, 49.8)	46.6 (45.2, 48.2)	0.51	44.4 (41.8, 47.2)	44.3 (42.3, 46.5)	0.95
LDL cholesterol level, mg/dL ^†^	112.7 (106.1, 119.7)	115.7 (111.0, 120.7)	0.48	108.0 (99.3, 117.4)	111.3 (105.4, 119.6)	0.30
Triglyceride level, mg/dL ^†^	137.6 (123.5, 153.2)	132.9 (123.2, 143.3)	0.60	125.7 (108.1, 146.0)	121.5 (108.4, 136.2)	0.62

lbs: pound; HbA1c: Hemoglobin A1c; HOMA-IR: insulin resistance index; HDL: high-density lipoproteins; LDL: low-density lipoprotein; Values are presented as mean 95% confidence intervals (95% CI). Multivariable adjusted: adjusted by sex, age, group, physical activity, energy, race, education; ^†^ A log transfer model was used to analyze these variables. MUFA: monounsaturated fatty acids; PUFA: polyunsaturated Fatty Acids; SFA: saturated Fatty Acids.

**Table 3:** Changes by breakfast eating pattern from baseline to one year.

	Breakfast Eating Pattern					
	Unadjusted			Multivariable Adjusted		
	Skipping (*n* = 21)	Eating (*n* = 117)	*p*	Skipping (*n* = 21)	Eating (*n* = 117)	*p*
**Physical characteristics**						
Body weight (lbs)	−6.5 (−9.2, −3.7)	−4.9 (−6.7, −3.1)	0.34	−6.6 (−11.3, −1.9)	−5.7 (−8.6, −2.9)	0.68
Waist circumference, in	0.0 (−0.5, 0.5)	−0.2 (−0.6, 0.1)	0.46	0.5 (−0.5, 1.4)	−0.2 (−0.8, 0.4)	0.16
BMI (kg/m^2^)	−9.6 (−12.7, −6.5)	−6.5 (−8.7, −4.4)	0.11	−1.0 (−3.7, 1.7)	−1.6 (−3.2, −0.0)	0.60
Blood pressure:						
Systolic (mmHg)	−1.8 (−4.5, 1.0)	−4.1 (−6.0, −2.3)	0.17	−5.3 (−10.1, −1.8)	−5.2 (−8.7, −1.6)	0.97
Diastolic (mmHg)	−2.7 (−4.8, −0.7)	−2.5 (−3.9, −1.1)	0.86	−5.9 (−6.2, 0.6)	−3.6 (−6.1, −1.1)	0.22
**Daily dietary intake**						
Total energy intake, kcal	−312.0 (−455.4, −168.6)	−365.0 (−458.9, −271.1)	0.54	−133.9 (−365.1, 97.4)	−260.7 (−398.1, −123.3)	0.23
Fat, %	−2.2 (−4.2, −0.1)	−2.9 (−4.3, −1.6)	0.54	2.7 (−1.0, 6.5)	−1.2 (−3.4, 1.1)	0.02
Protein, %	1.5 (0.2, 2.8)	1.9 (1.0, 2.7)	0.64	−0.2 (−2.5, 2.1)	1.6 (0.2, 3.0)	0.09
Carbohydrate, %	1.5 (−0.7, 3.7)	0.9 (−0.5, 2.3)	0.66	−1.4 (−5.6, 2.8)	−0.2 (−2.7, 2.3)	0.52
Fiber, g	5.3 (1.5, 9.1)	3.2 (1.6, 4.8)	0.30	9.0 (4.5, 13.6)	5.7 (3.0, 8.4)	0.11
Cholesterol, mg	−8.1 (−49.5, 33.3)	−69.5 (−96.6, −42.4)	0.02	−0.9 (−88.0, 86.2)	−57.2 (−108.9, −5.4)	0.15
MUFA, %	−0.4 (−1.4, 0.6)	−0.8 (−1.4, −0.1)	0.58	1.5 (−0.4, 3.4)	0.1 (−1.0, 1.3)	0.12
PUFA, %	0.0 (−0.8, 0.8)	−0.1 (−0.6, 0.5)	0.86	1.0 (−0.4, 2.4)	0.0 (−0.9, 0.8)	0.12
SFA, %	−1.7 (−2.7, −0.8)	−2.0 (−2.7, −1.4)	0.58	−0.1 (−1.7, 2.0)	−1.3 (−2.4, −0.2)	0.09
Added sugar, %	−19.3 (−32.8, −5.8)	−20.5 (−26.2, −14.8)	0.87	−25.5 (−41.9, −9.1)	−24.8 (−34.6 −14.9)	0.92
Sodium, mg	−208.3 (−680.3, −263.7)	−491.1 (−691.1, −291.1)	0.28	−36.2 (−593.6, 521.2)	−395.0 (−730.5, −59.4)	0.16
**Dietary quality scores**	4.6 (−0.8, 10.0)	5.8 (3.5, 8.1)	0.69	7.1 (0.3, 13.9)	7.8 (3.7, 11.8)	0.81
**Daily intake of selected micronutrient**						
Vitamin B_12_, µg	−0.1(−2.1, 1.9)	−0.1 (−0.9, 0.8)	0.97	−0.2 (−2.7, 2.3)	−0.1 (−1.6, 1.4)	0.96
Vitamin B_6_, mg	−0.1 (−0.5, 0.3)	0.0 (−0.2, 0.2)	0.58	0.1 (−0.4, 0.5)	0.1 (−0.2, 0.4)	0.88
Vitamin D, µg	0.5 (−1.5, 2.4)	−0.2 (−1.0, 0.6)	0.52	1.4 (−0.9, 3.8)	0.8 (−0.6, 2.2)	0.54
Vitamin E, mg	0.6 (−4.5, 5.6)	−1.6 (−3.7, 0.6)	0.44	4.4 (−1.7, 10.4)	2.1 (−1.5, 5.7)	0.41
Thiamin, mg	−0.2 (−0.6, 0.1)	−0.2 (−0.4, −0.1)	0.99	−0.2 (−0.6, 0.2)	−0.2 (−0.4, 0.0)	0.69
Calcium, mg	−79.4 (−223.2, 64.5)	−116.7 (−177.7, −55.8)	0.63	−16.9 (−185.0, 151.2)	−58.9 (−160.2, 42.4)	0.58
Magnesium, mg	4.7 (−37.3, 46.7)	1.0 (−16.8, 18.8)	0.87	42.2 (−5.6, 90.0)	24.1 (−4.7, 52.9)	0.40
Iron, mg	−0.9 (−3.9, 2.0)	−1.4 (−2.7, −0.1)	0.78	0.4 (−3.0, 3.8)	−0.5 (−2.6, 1.6)	0.54
Zinc, mg	−0.8 (−2.7, 1.1)	−1.0 (−1.8, −0.2)	0.86	0.4 (−2.0, 2.7)	−0.2 (−1.6, 1.2)	0.62
Niacin, mg	−1.7 (−5.2, 1.9)	−2.3 (−3.9, −0.8)	0.73	1.1 (−2.9, 5.2)	−0.5 (−2.9, 2.0)	0.38
Folate, µg	−8.5 (−104.3, 87.4)	−6.6 (−47.2, 34.0)	0.97	35.9 (−78.1, 149.9)	18.0 (−50.6, 86.7)	0.72
Riboflavin, mg	−0.2 (−0.5, 0.1)	−0.3 (−0.4, −0.2)	0.60	0.0 (−0.4, 0.3)	−0.2 (−0.4, 0.1)	0.44
Phosphorus, mg	−85.6 (−237.5, 66.2)	−115.9 (−180.3, −51.6)	0.72	28.3 (−144.3, 200.9)	−38.2 (−142.3, 65.8)	0.39
Potassium, mg	−108.6 (−460, 243.5)	−49.9 (−199.0, 99.3)	0.76	223.1 (−198.2, 644.3)	188.9 (−65.1, 442.8)	0.86
**Metabolic parameters**						
HbA_1c_ level, %	0.0 (−0.1, 0.1)	0.0 (−0.0, 0.0)	0.62	−0.1 (−0.2, 0.1)	−0.1 (−0.1, −0.0)	0.65
Fasting glucose level, mg/dL ^†^	2.3 (−2.8, 7.4)	1.7 (−0.5, 3.9)	0.81	−3.8 (−10.0, 2.4)	−3.0 (−6.8, 0.7)	0.79
Fasting plasma insulin level, mU/L ^†^	0.3 (−4.2, 4.7)	−4.0 (−6.0, −2.0)	0.08	−1.6 (−7.3, 4.1)	−5.5 (−9.1, −1.9)	0.14
HOMA-IR score ^†^	0.1 (−1.1, 1.4)	−0.9 (−1.5, −0.4)	0.12	−0.7 (−2.3, 0.9)	−1.6 (−2.6, 0.6)	0.22
**Blood lipid levels**						
Total cholesterol level, mg/dL ^†^	1.3 (−11.0, 13.7)	−3.9 (−9.3, 1.4)	0.44	−2.2 (−17.9, 13.5)	−6.0 (−15.5, 3.5)	0.59
HDL cholesterol level, mg/dL ^†^	−1.5 (−3.9, 1.0)	−0.2 (−1.3, 0.8)	0.36	−2.8 (−5.9, 0.3)	−1.3 (−3.2. 0.6)	0.29
LDL cholesterol level, mg/dL ^†^	4.1 (−6.7, 15.0)	−0.8 (−5.5, 4.0)	0.42	0.2 (−13.9, 13.5)	−3.4 (−11.8, 4.9)	0.60
Triglyceride level, mg/dL ^†^	−7.8 (−31.5, 16.0)	−14.7 (−25.0, −4.4)	0.60	2.5 (−27.5, 32.5)	−6.7 (−24.9, 11.6)	0.50

Values are presented as mean 95% confidence intervals (95% CI). Multivariable adjusted: adjusted by sex, age, group, physical activity, energy, race, education; ^†^ A log transfer model was used to analyze these variables. MUFA: monounsaturated fatty acids; PUFA: polyunsaturated Fatty Acids; SFA: saturated Fatty Acids.
